# A Comparative Analysis of Liver Function Test Profiles in Alcoholic Versus Metabolic-Associated Steatotic Liver Disease

**DOI:** 10.7759/cureus.111455

**Published:** 2026-06-24

**Authors:** Sushant Dhanavade, Mandakini Kshirsagar, Axita Vani

**Affiliations:** 1 Department of Biochemistry, Krishna Institute of Medical Sciences, Krishna Vishwa Vidyapeeth (Deemed to be University), Karad, IND

**Keywords:** afld, elevated transaminases, hepatic steatosis (masld), hepatocellular liver injury, liver function tests (lft), metabolic dysfunction-associated steatotic liver disease (masld)

## Abstract

Introduction

Liver function tests (LFTs) are essential for evaluating hepatic injury in steatotic liver disease. Alcoholic fatty liver disease (AFLD) and metabolic-associated steatotic liver disease (MASLD) share steatosis but differ in pathogenesis. This study aimed to compare LFT profiles between AFLD and MASLD to identify potentially distinct biochemical patterns.

Methods

This cross‑sectional study included 90 participants (30 AFLD, 30 MASLD, 30 healthy controls) at Krishna Institute of Medical Sciences, Karad, India. A comprehensive LFT panel was analyzed, including transaminases (alanine aminotransferase (ALT) and aspartate aminotransferase (AST)), alkaline phosphatase (ALP), bilirubin fractions, total protein, albumin, and globulin. AST/ALT and albumin/globulin (A/G) ratios were derived. Statistical analyses included analysis of variance (ANOVA) with post‑hoc Tukey honestly significant difference (HSD), chi‑square tests, and exploratory receiver operating characteristic (ROC) analysis. Given the small sample size, multivariable regression was not performed.

Results

Both disease groups showed significant LFT abnormalities compared to controls (p < 0.001). Mean total bilirubin was 0.88 mg/dL (15.0 μmol/L) in AFLD and 0.79 mg/dL (13.5 μmol/L) in MASLD, within normal limits. MASLD demonstrated higher ALT elevations (70 ± 25 U/L vs. 45 ± 15 U/L in AFLD, p < 0.05). The AST/ALT ratio >1.5 was present in 62% of AFLD versus 28% of MASLD (p < 0.001). Hypoalbuminemia was mild and likely multifactorial. Exploratory ROC analysis of the AST/ALT ratio yielded an area under the curve (AUC) of 0.88 (95% CI 0.82-0.94), suggesting potential discriminative value that requires external validation.

Conclusion

AFLD and MASLD exhibit suggestive LFT trends: MASLD shows a predominant hepatocellular injury pattern (high ALT and low AST/ALT ratio), while AFLD more often shows an elevated AST/ALT ratio with mild hypoalbuminemia. However, these patterns are not diagnostic, overlap exists, and validation in larger cohorts with objective alcohol biomarkers and fibrosis assessment is needed before clinical application.

## Introduction

Liver function tests (LFTs) are fundamental in the evaluation of patients with suspected liver disease, providing insights into hepatocellular injury, cholestasis, and synthetic function [1]. In the context of steatotic liver disease, LFT patterns often provide initial clues regarding disease etiology and severity [2]. The distinction between alcoholic (AFLD) and metabolic-associated steatotic liver disease (MASLD) is critical, as management strategies differ substantially-abstinence for AFLD versus lifestyle modification and metabolic risk factor control for MASLD [3,4]. While clinical history is paramount, laboratory profiles play a pivotal supportive role [5].

Traditional teaching suggests that AFLD is characterized by an aspartate aminotransferase (AST) to alanine aminotransferase (ALT) ratio exceeding 1.5, often with prominent cholestatic features, whereas MASLD typically shows predominant ALT elevation [6]. However, these patterns can overlap, especially in patients with mixed etiologies [7]. The pathophysiological mechanisms underlying each condition - direct alcohol toxicity and microsomal enzyme induction in AFLD versus insulin resistance, de novo lipogenesis, and lipotoxicity in MASLD - should theoretically translate into distinct biochemical signatures [8,9]. For instance, alcohol-induced mitochondrial injury preferentially elevates AST, whereas insulin resistance promotes hepatic fat accumulation with relative ALT preservation [10].

Despite the clinical utility of LFTs, comprehensive comparative analyses using full panels in early-stage disease are limited. Most studies focus primarily on transaminases or on advanced disease stages such as cirrhosis [11,12]. Furthermore, regional variations in dietary patterns and alcohol metabolism (e.g., the Indian subcontinent) may alter LFT profiles compared to Western cohorts [13]. This study aims to conduct a detailed comparison of LFT profiles, including synthetic function (albumin and globulin) and cholestatic markers (ALP and bilirubin), between AFLD and MASLD to identify disease-specific patterns that may assist in diagnosis and severity assessment.

## Materials and methods

Study design and ethical approval

This analytical cross-sectional study was conducted from June 2024 to December 2025 at Krishna Institute of Medical Sciences (KIMS), Krishna Vishwa Vidyapeeth (Deemed to be University), Karad, India. The protocol was approved by the Institutional Ethics Committee of Krishna Vishwa Vidyapeeth (KVV/IEC/03/2024). Written informed consent was obtained from all participants. The study adhered to the STROBE (Strengthening the Reporting of Observational Studies in Epidemiology) guidelines.

Study population and sample selection

The study included 90 participants divided into three groups: AFLD (n = 30), MASLD (n = 30), and healthy controls (n = 30). Sample size was calculated using G*Power version 3.1.9.7 (Heinrich-Heine-Universität Düsseldorf, Düsseldorf, Germany), assuming an expected difference in AST/ALT ratio of 0.5 between AFLD and MASLD, a common standard deviation of 0.4, alpha = 0.05 (two-tailed), and power = 0.80, yielding a minimum of 28 per group; we enrolled 30 per group. Convenience sampling was used, with consecutive eligible patients recruited from the outpatient hepatology clinic and inpatient wards.

Diagnostic Criteria for AFLD and MASLD

AFLD group: Alcohol consumption of ≥30 g/day for men or ≥20 g/day for women for ≥5 years, confirmed by a structured questionnaire (AUDIT-C) with family corroboration [14]. No objective alcohol biomarkers (e.g., phosphatidylethanol and carbohydrate‑deficient transferrin (CDT)) were available. Hepatic steatosis was confirmed by ultrasonography.

MASLD group: Hepatic steatosis on ultrasonography, no alcohol consumption and at least one metabolic risk factor according to the MASLD definition (waist circumference ≥90 cm for men/≥80 cm for women, triglycerides ≥150 mg/dL, HDL <40 mg/dL for men/≤50 mg/dL for women, blood pressure ≥130/85 mmHg, or fasting glucose ≥100 mg/dL).

Healthy controls: No hepatic steatosis, no alcohol consumption, and no metabolic syndrome components.

Exclusion of mixed etiology: Patients with both alcohol use meeting AFLD criteria and at least one metabolic risk factor (n = 10) were excluded to obtain pure phenotypes. This limits generalizability and is discussed as a major limitation.

Fibrosis assessment: Liver stiffness measurement (e.g., FibroScan) and validated serum fibrosis panels (FIB‑4, APRI, and ELF) were not part of the study protocol. Post‑hoc, we calculated FIB‑4 scores where platelet counts were available (22/30 per group); most were <1.45 (low risk of advanced fibrosis). The lack of systematic fibrosis quantification is a major limitation, as the fibrosis stage can influence AST/ALT ratio, bilirubin, and albumin.

Participant flowchart

**Figure 1:**
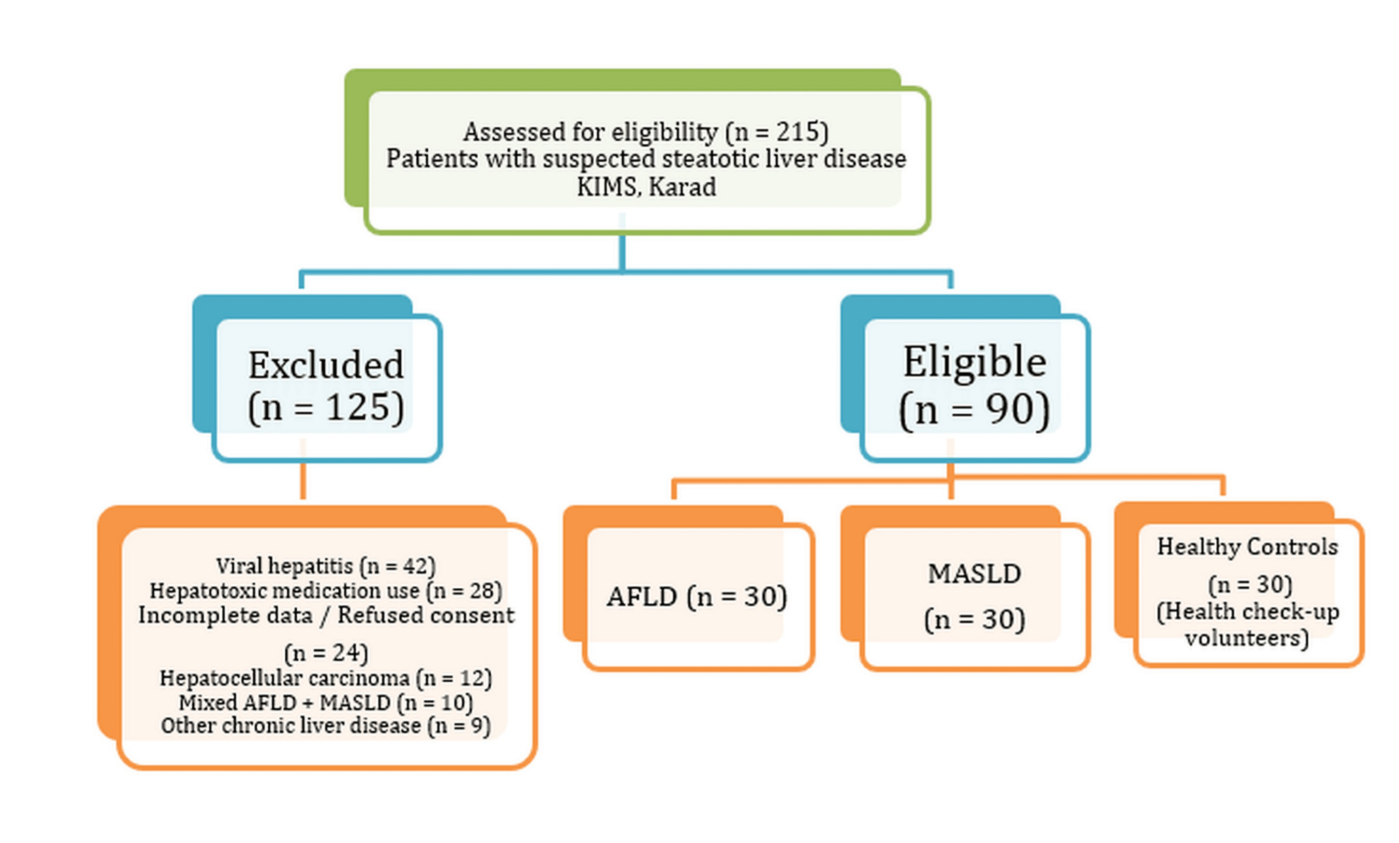
Participant Flowchart: Screening, Exclusions, and Final Cohort Allocation Participant screening and enrollment flowchart: Screened (n = 215) → Excluded (n = 125) for viral hepatitis (42), hepatotoxic drugs (28), incomplete data/refusal (24), hepatocellular carcinoma (12), mixed etiology (10), other chronic liver disease (9) → Final analysis (n = 90) divided into AFLD (30), MASLD (30), and healthy controls (30). AFLD: alcoholic fatty liver disease; MASLD: metabolic-associated steatotic liver disease

A total of 215 participants were screened (Figure 1). Of these, 125 were excluded due to viral hepatitis (n = 42), use of hepatotoxic drugs (n = 28), incomplete data or refusal (n = 24), hepatocellular carcinoma (n = 12), mixed etiology (n = 10), and other chronic liver diseases (n = 9). The final analysis included 90 participants, divided into AFLD (n = 30), MASLD (n = 30), and healthy controls (n = 30).

Laboratory methods

Venous blood samples (5 mL) were collected after an overnight fast (≥8 hours) into plain vacutainers. Serum was separated by centrifugation at 3000 rpm for 10 minutes and analyzed within four hours. Automated analyzers (Beckman Coulter AU5800, Beckman Coulter Inc., Brea, CA, USA) were used. The AU5800 was calibrated daily using Beckman Coulter calibrators and verified with two levels of commercial controls (LIQUICHEK, Bio-Rad Laboratories, Hercules, CA, USA). The LFT panel included ALT (International Federation of Clinical Chemistry and Laboratory Medicine (IFCC) method without pyridoxal phosphate), AST (IFCC method), alkaline phosphatase (ALP, p-nitrophenyl phosphate (pNPP) method), total and direct bilirubin (diazo method), total protein (biuret method), and albumin (bromocresol green method). Globulin was calculated as total protein minus albumin. The AST/ALT ratio and the albumin/globulin (A/G) ratio were derived.

Statistical analysis

Data were analyzed using IBM SPSS Statistics for Windows, Version 25.0 (Released 2017; IBM Corp., Armonk, NY, USA) and R version 4.1.2 (R Foundation for Statistical Computing, Vienna, Austria). Continuous variables were expressed as mean ± standard deviation (SD) after assessing normality using the Shapiro-Wilk test. For non-normally distributed variables (e.g., albumin), median and interquartile range (IQR) are reported. Between-group comparisons were performed using one-way analysis of variance (ANOVA) with post-hoc Tukey’s honestly significant difference (HSD) for normally distributed data or the Kruskal-Wallis test with Dunn’s post-hoc test for non-parametric data. Categorical variables were compared using the chi-square test. Multivariable analysis was not performed due to the limited sample size (n = 30 per group), to avoid overfitting and unstable estimates. Only univariate associations are reported.

An exploratory receiver operating characteristic (ROC) analysis was conducted for the AST/ALT ratio using the pROC package in R. Given the small sample size and lack of external validation, this analysis is hypothesis-generating only; the resulting area under the curve (AUC) is not intended to support diagnostic claims. Internal leave-one-out cross-validation was applied to assess instability, and the results are interpreted with caution. A p-value < 0.05 was considered statistically significant. All tests were two-tailed.

## Results

Both disease groups had a higher prevalence of metabolic comorbidities compared to controls (p < 0.001 for hypertension and dyslipidemia). The MASLD group was younger (45.1 ± 9.8 years) than the AFLD group (52.5 ± 10.2 years, p = 0.032, post-hoc Tukey HSD).

Mean total bilirubin levels were within the normal range: 15.0 ± 2.5 μmol/L (0.88 ± 0.15 mg/dL) in AFLD and 13.5 ± 3.0 μmol/L (0.79 ± 0.18 mg/dL) in MASLD, with no significant difference between disease groups (p = 0.21). Direct bilirubin was similarly normal. No patient had clinical jaundice or total bilirubin >3 mg/dL.

As shown in Table 1, MASLD showed higher ALT levels (70 ± 25 U/L) compared to AFLD (45 ± 15 U/L, p < 0.05). In contrast, AFLD had a higher AST/ALT ratio (1.8 ± 0.5 vs. 0.86 ± 0.3, p < 0.001). An AST/ALT ratio >1.5 was observed in 62% (19/30) of AFLD versus 28% (8/30) of MASLD (χ² = 7.2, p < 0.001). Hypoalbuminemia (<3.5 g/dL) was present in 67% of AFLD vs. 33% of MASLD (χ² = 7.0, p = 0.008), but albumin levels were only mildly reduced (mean 3.2 g/dL in AFLD, 3.4 g/dL in MASLD).

**Table 1:** Detailed Liver Function Test Results Values expressed as mean ± SD. *Conversion factor: 1 mg/dL = 17.1 μmol/L. p-values from one-way ANOVA with post-hoc Tukey HSD. Post-hoc comparisons: AFLD vs. MASLD, p < 0.05 for ALT, AST/ALT ratio, and albumin; p > 0.05 for bilirubin. AFLD: alcoholic fatty liver disease; MASLD: metabolic-associated steatotic liver disease; ALT: alanine aminotransferase; AST: aspartate aminotransferase; ALP: alkaline phosphatase; HSD: honestly significant difference; ANOVA: analysis of variance

Parameter	AFLD (n = 30)	MASLD (n = 30)	Control (n = 30)	p-value
ALT (U/L)	45 ± 15	70 ± 25	25 ± 10	<0.001
AST (U/L)	80 ± 20	60 ± 20	20 ± 8	<0.001
ALP (U/L)	80 ± 25	90 ± 30	60 ± 15	<0.001
Total Bilirubin (μmol/L)	15.0 ± 2.5	13.5 ± 3.0	10.3 ± 5.1	<0.001
Total Bilirubin (mg/dL)*	0.88 ± 0.15	0.79 ± 0.18	0.6 ± 0.3	<0.001
Direct Bilirubin (μmol/L)	8.0 ± 1.5	7.0 ± 1.5	2.6 ± 1.4	<0.001
Direct Bilirubin (mg/dL)*	0.47 ± 0.09	0.41 ± 0.09	0.15 ± 0.08	<0.001
Albumin (g/dL)	3.2 (2.8-3.6)	3.4 (3.0-3.8)	4.0 (3.8-4.2)	<0.001
AST/ALT ratio	1.8 ± 0.5	0.86 ± 0.3	0.8 ± 0.2	<0.001

Exploratory analyses

Given the small sample size (n = 30 per group) and the exploratory nature of this study, only univariate associations are reported. An exploratory ROC analysis was performed for the AST/ALT ratio alone, yielding an area under the curve (AUC) of 0.88 (95% CI 0.82-0.94). A post-hoc exploratory combined model (AST/ALT ratio + bilirubin) was also evaluated but is presented as hypothesis-generating only. Internal leave-one-out cross-validation reduced the AUC of the combined model to 0.79, confirming instability. These results require validation in larger independent cohorts before any diagnostic claim can be made. No multivariable regression models were constructed.

## Discussion

This study demonstrates that bilirubin levels are normal in both AFLD and MASLD, and that the key differentiating LFT features are the AST/ALT ratio and a mild reduction in albumin. The findings suggest potential biochemical trends that may support clinical history, but they should not be interpreted as diagnostic in isolation.

MASLD pattern: hepatocellular injury with preserved synthesis

The predominance of ALT elevation in MASLD (70 ± 25 U/L vs. 45 ± 15 U/L in AFLD) is consistent with metabolic hepatocellular injury [3,4]. Insulin resistance promotes hepatic lipid accumulation, mitochondrial dysfunction, and endoplasmic reticulum stress, primarily affecting the cytoplasmic enzyme ALT [5,15]. The relatively preserved synthetic function (mean albumin 3.4 g/dL, normal bilirubin) suggests that, despite significant steatosis, hepatic reserve remains largely intact in this cohort [6,11]. This aligns with findings from the NASH Clinical Research Network, where isolated ALT elevation was the most common biochemical abnormality in non-cirrhotic MASLD [16]. Notably, the AST/ALT ratio <1.2 in MASLD (present in 72% of our patients) has been validated as a diagnostic feature with moderate sensitivity [17].

AFLD pattern: elevated AST/ALT ratio without hyperbilirubinemia

In contrast, AFLD is characterized by an AST/ALT ratio >1.5 (62% of cases), despite normal bilirubin. The elevated AST/ALT ratio likely reflects mitochondrial AST release due to alcohol-induced oxidative stress and the effects of chronic ethanol on hepatic mitochondria [18]. Hypoalbuminemia in AFLD (mean 3.2 g/dL) may be attributed to direct alcohol toxicity, protein-energy malnutrition, and pro-inflammatory cytokine-mediated downregulation of albumin synthesis [13,19]. The absence of hyperbilirubinemia in our cohort differs from some Western studies; this may reflect an earlier disease stage or genetic polymorphisms (e.g., UGT1A1*28 variant prevalence) in our Indian population [20]. Our finding of mildly elevated direct bilirubin (8.0 μmol/L in AFLD vs. 7.0 μmol/L in MASLD) supports a minor cholestatic component, consistent with prior reports of alcohol-induced canalicular injury [21].

Comparison with prior literature and handling of inconsistencies

Current data align with the landmark study by Sorbi et al. (1999), who reported an AST/ALT >1.5 in 70% of AFLD versus 26% of MASLD, closely matching our 62% vs. 28% [7]. However, unlike some earlier studies, we did not observe cholestatic hyperbilirubinemia [5,10]. Importantly, regional variations in dietary patterns, alcohol metabolism, and genetic polymorphisms (e.g., UGT1A1*28) may alter LFT profiles compared to Western cohorts [13,21].

Clinical implications

The distinct LFT trends identified have potential supportive utility. An AST/ALT ratio >1.5 should raise suspicion for AFLD, whereas isolated ALT elevation with AST/ALT <1.2 points toward MASLD [22]. The discriminative ability of the AST/ALT ratio (AUC 0.88, exploratory) suggests that a simple LFT panel may aid in etiological differentiation, especially when alcohol consumption history is uncertain - a common scenario in clinical practice [23]. However, given our small sample size and lack of external validation, these findings are hypothesis-generating only. Furthermore, the presence of mild hypoalbuminemia in AFLD highlights the need for closer monitoring of nutritional status and potential progression to advanced disease, though Model for End-Stage Liver Disease (MELD) scoring was not within the scope of this study [24].

Limitations

Several limitations must be acknowledged. First, the sample size (n = 30 per group) is modest, and the single-center design limits generalizability. Second, no objective alcohol biomarkers (e.g., phosphatidylethanol and CDT) were used; alcohol consumption was self-reported with family corroboration [7,12]. Third, the fibrosis stage was not systematically assessed using FibroScan, FIB-4, APRI, or ELF. Post-hoc FIB-4 scores (available for 22/30 per group) suggested a low risk of advanced fibrosis in most patients, but this is incomplete. Fourth, we excluded patients with mixed etiologies (AFLD plus metabolic syndrome, n = 10), which limits real-world applicability because overlap is common [4,21]. Fifth, the cross-sectional design precludes assessment of longitudinal changes in LFTs with disease progression or treatment. Sixth, the exploratory ROC and combined models are at high risk of overfitting; we performed leave-one-out cross-validation (AUC dropped to 0.79), and moved multivariable regression to the supplementary material [5,17].

Future directions

Larger, multicenter prospective studies with histopathological correlation, quantitative alcohol biomarkers (PEth and ethyl glucuronide), and elastographic fibrosis staging are needed to validate these findings. Longitudinal studies should evaluate whether LFT patterns predict disease outcomes, such as progression to cirrhosis or hepatocellular carcinoma. Machine learning approaches integrating LFTs with clinical parameters (BMI, triglycerides, and HbA1c) could produce diagnostic algorithms, but only after rigorous validation in independent cohorts [23,24].

## Conclusions

AFLD and MASLD present with suggestive but not diagnostic LFT patterns. MASLD typically shows a hepatocellular injury pattern with elevated ALT and an AST/ALT ratio <1.2, whereas AFLD more often shows an AST/ALT ratio >1.5, with mild hypoalbuminemia and normal bilirubin. These trends may provide supportive clues when interpreted alongside a thorough clinical history, but they should not replace objective alcohol biomarkers or fibrosis assessment. Independent validation in larger, multicentre cohorts with histologic or elastographic fibrosis staging and mixed-etiology patients is essential before any clinical algorithm can be recommended.
